# Clinical, Physiologic, and Anatomic Outcomes of a Novel Bioprosthetic Aortic Valved Conduit

**DOI:** 10.3390/jcm15093437

**Published:** 2026-04-30

**Authors:** Sedem Dankwa, Ely Erez, Adrian R. Acuna Higaki, Shiv Verma, Irbaz Hameed, Sriharsha Talapaneni, Kristina Wang, Sem Asmelash, Titilayo Oden Shobayo, Pavan Khosla, Kwasi Ansere Ofori, Roland Assi, Prashanth Vallabhajosyula

**Affiliations:** Division of Cardiac Surgery, Department of Surgery, Yale University School of Medicine, New Haven, CT 06520, USA

**Keywords:** thoracic aortic aneurysm, aortic root replacement, bio-Bentall procedure, bioprosthetic aortic valved conduit, aortic hemodynamics

## Abstract

**Background:** In 2020, the first pre-assembled bioprosthetic aortic valved conduit (AVC) was approved in the United States. This study compares its anatomic and functional outcomes to traditional hand-sewn composite conduits in patients undergoing aortic root replacement. **Methods:** This retrospective study compared 118 patients receiving the pre-assembled AVC (2021–2023) versus 66 patients with hand-sewn conduits (2012–2020) after elective bio-Bentall procedures. Primary outcomes were post-operative mortality and complication rates. Secondary outcomes included anatomic and hemodynamic changes. Graft dimensions were obtained from post-operative computed tomography (CT). Echocardiographic parameters were collected at early and late follow-up. Between-group differences and longitudinal changes were assessed using linear mixed-effects models. **Results:** Groups were comparable in age (pre-assembled 63 ± 11 vs. hand-sewn 64 ± 11 years) and predominantly male. Despite significantly higher concomitant hemiarch rates in pre-assembled conduits (91.5% vs. 28.8%, *p* < 0.001), 30-day mortality, stroke, and reoperation for bleeding were comparable between groups. Pre-assembled conduits demonstrated superior hemodynamics with lower baseline peak gradients (Δ 9.1 mmHg, *p* < 0.001), lower mean gradients (Δ 5.3 mmHg, *p* < 0.001), and larger indexed effective orifice area (Δ 0.27 cm^2^/m^2^, *p* = 0.018). Annual rates of hemodynamic and dimensional change were minimal and comparable between groups. Kaplan–Meier analysis showed no survival difference at 3 years. **Conclusions:** The pre-assembled AVC demonstrates equivalent safety and superior early hemodynamic performance compared to hand-sewn conduits, with stable mid-term anatomic and functional outcomes.

## 1. Introduction

Since its introduction in 1968, the Bentall procedure has been a cornerstone in the treatment of aortic root pathologies in patients with concomitant aortic valve disease [1]. Traditionally, younger patients receive mechanical valves for durability and survival advantage [2], but improvements in bioprosthetic valve longevity [3], along with the potential for valve-in-valve transcatheter aortic valve replacement (TAVR) in cases of valvular degeneration [4,5], have led to an increased use of bioprosthetic valves in Bentall procedures [6]. Bio-Bentall conduit options include stentless bioprostheses such as the Medtronic Freestyle (Medtronic, Minneapolis, MN), porcine root xenografts [7], which are often extended using a synthetic aortic graft, and stented bioprostheses, which are hand-sewn into a synthetic aortic graft to create a composite conduit during the procedure [8].

Despite their increasing use, both stentless and hand-sewn stented bioprostheses have notable drawbacks, including variability in graft construction and extended operative times. In 2020, KONECT RESILIA (Edwards Lifesciences, Irvine, CA, USA), the first pre-assembled aortic valved conduit (AVC), was developed to address these issues. This device comprises a stented bovine pericardial trileaflet valve on a flexible frame, pre-assembled to a gelatin-impregnated, woven polyester Valsalva graft. Although early clinical reports have shown promising results, the available data remain limited [9,10]. We compare short- to early mid-term outcomes in patients who underwent aortic root replacement using the pre-assembled AVC versus traditional hand-sewn composite conduits.

## 2. Patients and Methods

### 2.1. Study Design and Population

This single-center, retrospective cohort study compared adult (18 years and older) patients who underwent elective bio-Bentall procedures using the first FDA-approved pre-assembled AVC versus traditional hand-sewn composite conduits at Yale New Haven Hospital, Connecticut. The pre-assembled cohort included operations between 2021 and 2023, while the hand-sewn cohort comprised patients treated between 2012 and 2020. Emergency cases were excluded. Follow-up extended through June 2024. This study was approved by the Institutional Review Board (IRB) under protocol number 2000020356.

### 2.2. Data Collection

Patient demographics, medical history, operative details, and morbidity and mortality data were extracted from electronic medical records. Graft dimensions were manually measured on initial post-operative and most recent computed tomography scans. Bioprosthetic valve function and cardiac parameters were assessed by means of echocardiography post-operatively and at 6, 12, and 18 months, selecting the nearest study within a 3-month window for each time point.

### 2.3. Operative Technique

Patients were placed on cardiopulmonary bypass (CPB) via central aortic, right atrial, and superior vena cava cannulation. In cases of concomitant transverse hemiarch replacement, patients were cooled to deep hypothermia (18–20 °C) to achieve electroencephalographic silence. Myocardial protection was achieved with antegrade and retrograde cold blood with Del Nido (1:3 parts) cardioplegia, administered at regular intervals throughout the cross-clamp period. After the aortic cross clamp was placed, the ascending aorta was resected above the sinotubular junction, the aortic valve was resected, and the annulus was decalcified. The aortic root was circumferentially dissected, the sinuses of Valsalva were resected, and the left and right coronary artery buttons were tailored. Aortic root sutures were placed in supra-annular interrupted fashion using 2-0 Ti-Cron pledgeted sutures. Root sutures were passed through the root conduit cuff and tied down. Left and right coronary artery buttons were anastomosed to the respective neo sinuses of the root conduit. Transverse hemiarch anastomosis was typically performed under deep hypothermic circulatory arrest with retrograde cerebral perfusion. Upon completion, CPB was re-initiated via arch graft arterial cannulation and the patient was re-warmed to come off CPB.

### 2.4. Outcomes

Primary outcomes of this study included operative mortality (in-hospital/30-day), late mortality (after 30 days), length of hospital stay, aortic reoperations, and complications during index hospitalization including stroke, renal failure necessitating temporary dialysis, extracorporeal membrane oxygenation (ECMO) support, takeback for bleeding, and implantation of a permanent pacemaker. Secondary outcomes included anatomic changes in graft dimensions on follow-up CT imaging, as well as functional and anatomic changes determined during echocardiographic follow-up.

### 2.5. Statistical Analysis

Continuous variables are reported as means ± standard deviations or medians with interquartile ranges, as appropriate. Categorical variables are expressed as frequencies and percentages. Missing data were assumed to be missing at random, and no imputation was performed. Longitudinal hand-sewn follow-up was limited to time points matching the pre-assembled group to ensure fair comparison.

Longitudinal changes in CT-derived aortic dimensions and echocardiographic parameters were analyzed using linear mixed-effects models accounting for repeated measures within subjects [11]. Models included a random intercept for patient-level variability and fixed effects for conduit type (pre-assembled vs hand-sewn), time (years from surgery), and their interaction. Baseline differences represent estimated early post-operative differences between groups. The yearly change coefficient reflects the annual rate in the hand-sewn group, while the interaction term indicates differences in the annual rate of change between the two groups.

Survival was estimated using Kaplan–Meier methods and compared with the log-rank test. Statistical significance was defined as *p* < 0.05. Analyses were performed using R Statistical Software (v4.3.3; R Core Team 2024).

## 3. Results

We evaluated 118 patients receiving a novel pre-assembled AVC against a reference group of 66 patients with traditional hand-sewn composite conduits after elective Bentall aortic root replacement. Demographic and clinical characteristics of the study cohort are summarized in Table 1. Groups were comparable in age (pre-assembled 63.2 ± 11.3 vs. hand-sewn 64.4 ± 10.5 years, *p* = 0.50) and predominantly male (pre-assembled 108 [91.5%] vs. hand-sewn 60 [90.9%], *p* = 0.89). Hypertension was the most common comorbidity (pre-assembled 73 [61.9%] vs. hand-sewn 51 [77.3%]), and both groups had similar rates of prior aortic surgery (pre-assembled 5 [4.2%] vs. hand-sewn 2 [3.0%], *p* = 0.73).

Across perioperative domains, groups showed broadly similar profiles, with the main differences in rates of aortic aneurysm, hemiarch repair, and valve size selection (Table 2). The most common indications for surgery were aortic aneurysm (pre-assembled 116 [98.3%] vs. hand-sewn 58 [87.9%], *p* = 0.01) and aortic stenosis (16 [13.6%] vs. 11 [16.7%], *p* > 0.1). A total of 108 patients (91.5%) in the pre-assembled group underwent concomitant hemiarch repair with hypothermic circulatory arrest compared to 19 (28.8%) in the hand-sewn group (*p* < 0.001), while rates of concomitant CABG were similar (23 [19.5%] vs. 11 [16.7%], *p* > 0.1). Consequently, the pre-assembled group had longer median cardiopulmonary bypass (262 [216–308] min vs. 162 [148–190] min, *p* < 0.001) and cross-clamp times (203 [170–240] min vs. 126 [108–145] min, *p* < 0.001) but similar circulatory arrest times (18 [15–21] min vs. 22 [18–29] min, *p* = 0.18).

Despite similar body surface area between groups (pre-assembled 2.13 ± 0.24 m^2^ vs. hand-sewn 2.13 ± 0.29 m^2^, *p* = 0.97), larger valves were selected more frequently in the pre-assembled group (e.g., 27 mm: 53 [44.9%] vs. 6 [9.1%], *p* < 0.001; Table 2). Median hospitalization was comparable (6.5 days [IQR 5–8] vs. 6.0 days [IQR 5–8], *p* = 0.42). Median follow-up was 28.2 months (IQR 23.5–38.9) in the pre-assembled group and 89.6 months (IQR 65.8–104.5) in the hand-sewn group, with outcome comparisons limited to 3 years.

**Figure 1:**
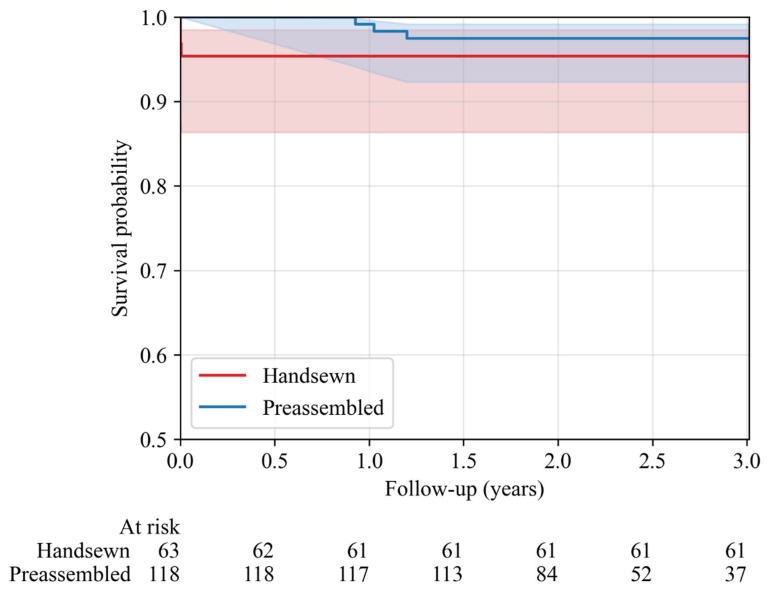
Kaplan–Meier estimates of three-year survival following bio-Bentall by conduit type.

Operative mortality, stroke, and takeback for bleeding within 30 days were similar between groups (*p* > 0.1), as were major complication rates including ECMO, temporary dialysis, and pacemaker implantation (*p* > 0.1) (Table 2). Kaplan–Meier analysis showed excellent, equivalent survival at 3 years (Figure 1).

**Figure 2:**
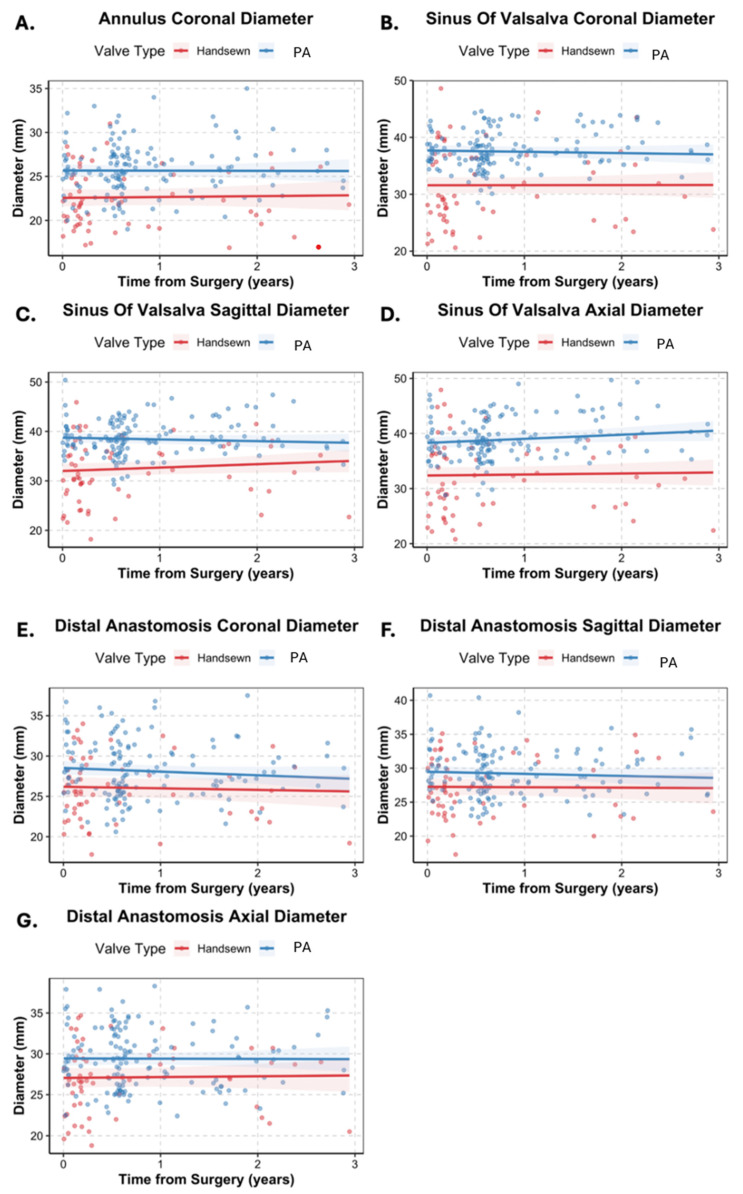
Three-year CT aortic diameter changes by conduit type. Lines represent estimates from linear mixed-effects models with 95% confidence intervals. Panels: (**A**) annulus diameter in the coronal plane, (**B**) sinus of Valsalva (SoV) diameter in the coronal plane, (**C**) SoV diameter in the sagittal plane, (**D**) SoV diameter in the axial plane, (**E**) distal anastomosis (DA) diameter in the coronal plane, (**F**) DA diameter in the sagittal plane, (**G**) DA diameter in the axial plane.

Longitudinal CT and echocardiographic measurements showed preserved aortic dimensions and valve performance in both cohorts across early and late follow-up (Table 3). CT-derived aortic diameters were measured at early (pre-assembled 203 [122–244] days vs. hand-sewn 65 [41–142] days) and late (pre-assembled 576 [244–722] days vs. hand-sewn 519 [153–769] days) post-operative time points. In the coronal plane, neosinus of Valsalva diameters for the pre-assembled AVC measured 37.63 ± 3.27 mm initially and 37.97 ± 3.38 mm at the latest follow-up, while hand-sewn diameters measured 31.37 ± 6.89 mm and 31.11 ± 6.54 mm, respectively. Similar stability was observed for annular and distal anastomosis diameters (Table 3). At baseline, pre-assembled AVCs were larger at the annulus (Δ 3.1 mm [95% CI 1.9–4.3], *p* < 0.001), neosinuses of Valsalva (Δ 6.1 mm [4.3–7.9], *p* < 0.001), and distal anastomosis (Δ 2.3 mm [0.8–3.8], *p* < 0.01) in the CT coronal plane, with similar findings in other planes (Table 4), consistent with more frequent use of larger valves in the pre-assembled group. Annual rate of change in aortic dimensions was minimal and comparable between groups (*p* > 0.05), indicating that dimensional differences were present early and remained stable throughout follow-up (Table 4, Figure 2).

**Figure 3:**
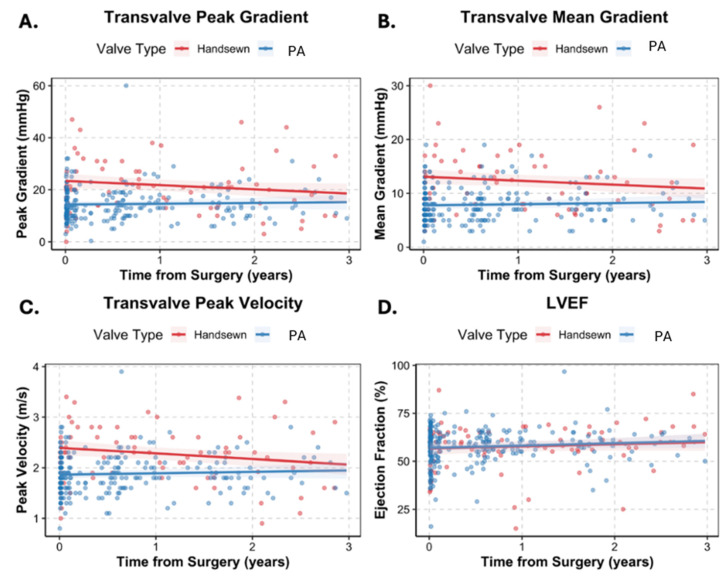

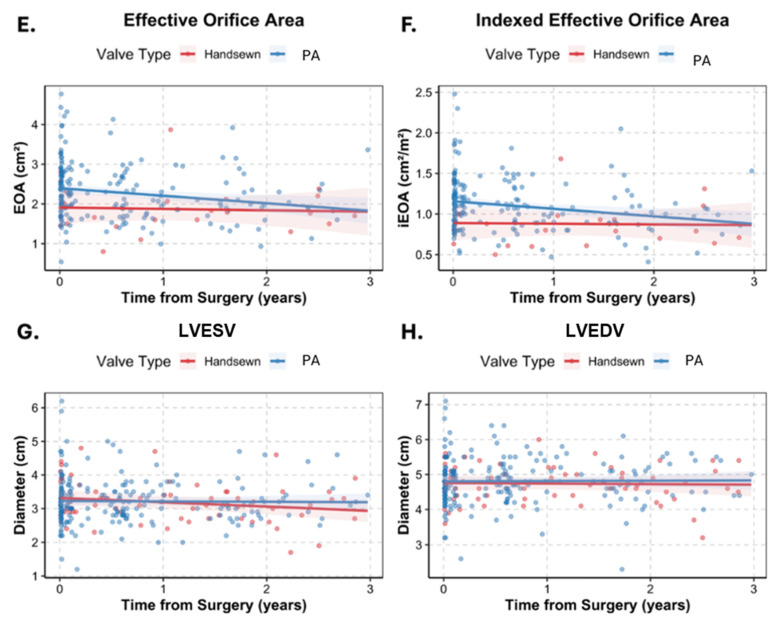
Three-year echocardiographic changes by conduit type. Lines represent estimates from linear mixed-effects models with 95% confidence intervals. Panels: (**A**) transvalve peak gradient, (**B**) transvalve mean gradient, (**C**) transvalve peak velocity, (**D**) left ventricular ejection fraction (LVEF), (**E**) effective orifice area (EOA), (**F**) indexed EOA (**G**) left ventricular end systolic volume (LVESV) and (**H**) left ventricular end diastolic volume (LVEDV).

Echocardiographic parameters for both groups were measured early (pre-assembled 6 [5–24] days vs. hand-sewn 60 [6–273] days) and late (pre-assembled 292 [195–626] days vs. hand-sewn 522 [326–770] days) post-operative follow-up time points. Across echocardiographic measures, linear mixed-effects model analysis demonstrated that AVC type was associated with meaningful differences, with modest parameter-specific time effects (summarized in Table 3 and Table 4 and illustrated in Figure 3). Overall, pre-assembled AVCs had lower Doppler velocities and gradients with larger valve areas at initial post-op measurement, with no differences in ventricular size or LVEF.

Echocardiographic parameters were measured early (pre-assembled 6 [5–24] days vs. hand-sewn 60 [6–273] days) and late (pre-assembled 292 [195–626] days vs. hand-sewn 522 [326–770] days) post-operatively. Overall, pre-assembled AVCs had lower Doppler velocities and gradients with larger valve areas at initial post-op measurement, with no differences in ventricular size or LVEF (Table 3 and Table 4, Figure 3). Pre-assembled AVCs demonstrated lower peak velocity (Δ 0.53 m/s [95% CI 0.36–0.70], *p* < 0.001), peak gradient (Δ 9.1 mmHg [6.0–12.1], *p* < 0.001), and mean gradient (Δ 5.3 mmHg [3.8–6.8], *p* < 0.001) at baseline, with larger EOA (Δ 0.48 cm^2^ [0.05–0.92], *p* = 0.031) and indexed EOA (Δ 0.27 cm^2^/m^2^ [0.05–0.49], *p* = 0.018). Annual rates of change were minimal in both groups, with minimal interaction effects for peak velocity (Δ 0.14 [0.03–0.24] m/s per year, *p* = 0.012) and mean gradient (Δ 0.95 [0.03–1.86] mmHg per year, *p* = 0.04). There were no differences in LVEF or LV end-diastolic or end-systolic volumesat baseline or over time (*p* > 0.05).

## 4. Comment

In this retrospective comparative study, we evaluated 118 patients receiving a novel pre-assembled AVC against 66 patients with traditional hand-sewn composite conduits after elective Bentall aortic root replacement. Under elective operative conditions, the pre-assembled AVC demonstrated excellent and comparable safety outcomes with superior early hemodynamic performance relative to hand-sewn conduits, while both approaches maintained stable anatomic aortic dimensions throughout follow-up.

Despite a higher prevalence of concomitant hemiarch replacement and CABG, the pre-assembled AVC cohort experienced no in-hospital or 30-day mortality, clinically meaningful stroke, or reoperation for bleeding, with major complication rates comparable to the hand-sewn reference group. Notably, the added complexity of circulatory arrest did not translate into increased morbidity or mortality. The longer cardiopulmonary bypass and cross-clamp times observed in the pre-assembled group are likely attributable to higher rates of hemiarch replacement, reflecting our institutional philosophy of aggressive prophylactic arch management in patients with ascending aortic aneurysms. This approach, facilitated by advances in cerebral perfusion strategies, represents standard institutional practice rather than a limitation of the pre-assembled AVC. Furthermore, differences in operative times may reflect changes in resident education platforms, including adoption of an I-6 cardiothoracic surgery training program over the past six years, which increased participation of junior residents in aortic root operations and likely contributed to longer operative times in the pre-assembled cohort; importantly, this did not negatively impact patient outcomes. An additional contributing factor may be that surgeons performing operations with the pre-assembled root conduit were not present at the institution during the earlier period when hand-sewn conduits were predominantly used.

A key finding of this study is the superior hemodynamic profile of the pre-assembled AVC compared to hand-sewn conduits. Pre-assembled conduits demonstrated significantly lower peak velocities (Δ 0.53 m/s), peak gradients (Δ 9.1 mmHg), and mean gradients (Δ 5.32 mmHg), along with larger effective orifice areas (Δ 0.48 cm^2^) and indexed EOA (Δ 0.27 cm^2^/m^2^). These differences were present at baseline and remained stable throughout follow-up, with minimal annual rates of change in both groups. Our preferential selection of larger valve sizes in the pre-assembled group compared to the reference hand-sewn cohort likely contributes to these hemodynamic differences. The valve sizing differences are again reflected in the larger absolute CT-measured aortic dimensions in the pre-assembled group. However, the demonstrated anatomic stability over time suggests the absence of pathologic ectasia in the early term. Further follow-up will help understand the hemodynamic functional consequences of the pre-assembled platform versus the hand-sewn platform.

Before the introduction of the pre-assembled AVC, biologic root replacement options were limited to stentless bioprostheses (e.g., Medtronic Freestyle) or hand-sewn stented composite conduits [12]. While both have demonstrated satisfactory outcomes [7,13], each has distinct limitations. Stentless bioprostheses provide favorable hemodynamics [14] and permit larger valve implantation [15] but are associated with higher rates of pseudoaneurysm formation [16], accelerated calcific degeneration [17,18], and more challenging reoperation [17]. Valve-in-valve TAVR is also more complex in stentless roots due to failure via aortic insufficiency with limited calcification for anchoring [19], absence of radiopaque markers [20], and increased coronary obstruction risk [21]. Additionally, their shorter length often necessitates extension grafts [7,12].

Stented composite conduits, by contrast, are not prone to root calcification or pseudoaneurysm formation, allow for a single distal anastomosis, and often permit valve replacement without complete conduit explantation in cases of degeneration [22], simplifying reoperation and valve-in-valve TAVR [20]. These advantages have contributed to the increasing preference for stented composite conduits in recent years [6,23]. However, hand-sewn composite conduits require intraoperative manual assembly, introducing variability in root geometry across surgeons and limiting comparability of outcomes. The pre-assembled AVC addresses these limitations by combining the advantages of stented composite conduits with a premanufactured design, eliminating manual assembly and providing more consistent anatomic and hemodynamic performance.

Thorough evaluation of new medical devices is essential, particularly for those classified as high risk by the FDA. As with transcatheter aortic valve replacement devices, composite aortic root conduits require ongoing evaluation to ensure long-term safety and effectiveness. While our study provides a detailed assessment of early outcomes with the pre-assembled AVC platform, additional studies and extended follow-up are required to further define its safety profile. Although no long-term data currently exist for the pre-assembled AVC itself, its individual components, the aortic valve and the Valsalva graft, are well established and have demonstrated favorable clinical performance. Beaver et al. reported favorable seven-year outcomes for the tissue valve, including stable hemodynamics, minimal regurgitation, and no evidence of structural valve deterioration [3]. Similarly, the Gelweave Valsalva graft has shown excellent results as part of hand-sewn composite conduits [13] and valve-sparing root replacement [24]. If the combined use of these validated components achieves comparable durability, the pre-assembled AVC may assume an important role in biologic aortic root replacement.

This would be timely given the growing preference for bio-Bentall procedures and the decline in mechanical valve use for Bentall operations [6]. These trends likely reflect an aging population with increasing aortic disease, greater awareness of complications associated with lifelong anticoagulation, improved durability of contemporary bioprosthetic valves [25], and the expanding success of valve-in-valve TAVR as an alternative to open redo surgery [4,5,20]. Adoption of pre-assembled bioprosthetic valved conduits may further accelerate this shift. It should be noted, however, that current evidence demonstrates a survival advantage for mechanical valves compared to bioprosthetic valves in patients under 60–65 years of age [2]. The higher cost of pre-assembled AVCs compared with hand-sewn conduits remains an important consideration for patients and aortic programs. Our experience demonstrates comparable early survival, safety, and anatomic stability between platforms, which may argue against routine use of a higher-cost conduit. However, preserved early-term hemodynamic superiority of the pre-assembled AVC, including lower gradients and larger EOA and indexed EOA, supports its selective use. Ultimately, longer-term follow-up is required to determine whether these differences persist in order to perform an authoritative cost–benefit analysis. Quite possibly, in patients of advanced age with a large aortic annulus, a stentless root or hand-sewn conduit may be more cost-effective without compromising outcomes. Similarly, in clinical scenarios such as endocarditis or when extensive root reconstruction is required, a more rigid system may be less optimal than a biologic, flexible alternative, including a homograft or Freestyle porcine xenograft conduit.

Our study has several limitations. First, the retrospective design and variability in patient follow-up resulted in missing echocardiographic reports at certain time points, potentially introducing bias. Although a linear mixed-effects model was utilized to address this concern, missing echocardiographic variables may not be missing at random, which could further contribute to bias. Second, the non-contemporaneous cohort design introduces potential confounding from evolving perioperative practices, institutional case mix, and surgeon experience over time. The marked difference in concomitant hemiarch utilization (91.5% vs. 28.8%) reflects this temporal shift in institutional practice rather than a device-specific effect, and readers should interpret between-group comparisons with this context in mind. Third, the preferential use of larger valve sizes in the pre-assembled cohort is an important source of confounding for hemodynamic comparisons. While pre-assembled conduits demonstrated lower gradients and larger effective orifice areas, these differences are at least partially attributable to systematic differences in implanted valve size rather than to device-specific hemodynamic properties alone. As such, size-stratified analyses in larger future cohorts will be needed to isolate device-specific effects. Fourth, the substantially shorter follow-up in the pre-assembled cohort (median approximately 28 months) compared to the hand-sewn cohort (median approximately 90 months) limits detection of late complications and precludes meaningful assessment of structural valve deterioration. Accordingly, conclusions regarding long-term durability cannot be drawn from the present dataset and must await extended surveillance. Finally, although data collection, analysis, and interpretation were performed independently of the operating surgeons who are co-authors of this manuscript, this should be recognized as a potential source of bias.

In summary, the early outcomes with the first FDA-approved pre-assembled AVC are promising, with low operative mortality, excellent hemodynamic performance, stable root anatomy, and minimal complications. These results suggest that the pre-assembled AVC is a reliable and effective choice for patients requiring a biologic aortic root and valve replacement. Longer follow-up will be essential to fully assess the conduit’s long-term durability and impact on patient outcomes, ultimately helping to shape the role of prefabricated bioprosthetic aortic valved conduits in aortic root replacement.

## Figures and Tables

**Table 1:** Demographic and clinical characteristics of the study cohort.

Characteristics	Pre-Assembled AVC (N = 118)	Hand-Sewn AVC (N = 66)	*p*-Value
Age, years	63.21 ± 11.33	64.35 ± 10.50	0.50
Male	108 (91.53)	60 (90.91)	0.89
Race/Ethnicity			
White	100 (84.75)	57 (86.37)	0.83
Black	8 (6.78)	6 (9.09)	0.57
Asian	1 (0.83)	0 (0)	1.00
Hispanic	2 (1.69)	0 (0)	1.00
Other	7 (5.93)	2 (3.03)	0.49
BMI, kg/m^2^	28.76 ± 5.21	29.02 ± 6.41	0.78
BSA, m^2^	2.13 ± 0.24	2.13 ± 0.29	0.97
Hypertension	73 (61.86)	51 (77.27)	0.03
Diabetes Mellitus	20 (16.95)	11 (16.67)	0.96
Dyslipidemia	33 (27.97)	35 (53.03)	<0.001
COPD	5 (4.24)	5 (7.58)	0.33
Chronic Kidney Disease	5 (4.24)	4 (6.06)	0.72
Active smoker	4 (3.39)	1 (1.52)	0.66
Cerebrovascular Disease	4 (3.39)	11 (16.67)	0.003
Peripheral Vascular Disease	1 (0.85)	2 (3.03)	0.29
Arrhythmia	11 (9.32)	12 (18.18)	0.08
Atrial Fibrillation	29 (24.58)	25 (37.88)	0.06
Coronary Artery Disease	19 (16.10)	7 (10.61)	0.30
Connective Tissue Disease	6 (5.08)	1 (1.52)	0.22
Bicuspid Aortic Valve	30 (25.42)	11 (16.67)	0.17
Prior MI	6 (5.08)	4 (6.06)	0.75
Prior PCI	5 (4.24)	0 (0.00)	0.16
Prior CABG	2 (1.69)	3 (4.55)	0.35
Prior Valve Surgery	6 (5.08)	2 (3.03)	0.62
Prior Aortic Surgery	5 (4.24)	2 (3.03)	0.73

**Table 2:** Operative characteristics and post-operative outcomes by conduit type.

Operative Characteristics	Pre-Assembled AVC (N = 118)	Hand-Sewn AVC (N = 66)	*p*-Value *
**Pre-operative**			
Indication for surgery			
Aortic Aneurysm	116 (98.31)	58 (87.85)	0.01
Aortic Reoperation	1 (0.85)	0 (0)	1.00
Aortic Stenosis	16 (13.56)	11 (16.67)	0.57
Aortic Dissection	3 (2.54)	4 (6.45)	0.23
Aortic Valve size			<0.001
19 mm	0 (0)	1 (1.52)	
21 mm	3 (2.54)	9 (13.64)	
23 mm	10 (8.47)	30 (45.45)	
25 mm	34 (28.81)	15 (22.73)	
27 mm	53 (44.92)	6 (9.09)	
29 mm	18 (15.25)	5 (7.58)	
**Intra-operative**			
Total operative time, min.	416.5 [350.75–472.75]	283.00 [255.50–358.00]	<0.001
CPB time, min.	262 [215.75–307.75]	162.00 [147.50–189.75]	<0.001
Cross clamp time, min.	203 [170.25–240]	126.39 [107.92–145.35]	<0.001
Hypothermic Circulatory Arrest	108 (91.52)	19 (28.79)	<0.001
Cerebral Perfusion	108 (91.52)	19 (28.79)	<0.001
Cerebral Perfusion time, min.	18 [15–21.25]	21.5 [18.25–29]	0.18
Concomitant operation			
Hemiarch	108 (91.52)	19 (28.79)	<0.001
MV repair	6 (5.08)	2 (3.03)	0.71
CABG	23 (19.49)	11 (16.67)	0.64
**Post-operative outcomes**			
Operative mortality	0 (0)	2 (3.00)	0.13
Stroke	0 (0)	1 (1.50)	0.36
Temporary dialysis	1 (0.85)	0 (0)	1.00
ECMO	1 (0.85)	0 (0)	1.00
Takeback for bleeding	0 (0)	1(1.50)	0.36
Permanent pacemaker implantation	3 (2.54)	2 (3.00)	1.00
Length of stay, days	6.5 [5–8]	6.0 [5–8]	0.42
Follow up, months	28.17 [23.53–38.86]	89.63 [65.77–104.47]	<0.001

* *p*-values were calculated using Student’s *t*-test for continuous variables, Chi-squared or Fisher’s exact test for categorical variables, and Wilcoxon rank-sum test for ordinal variables, as appropriate. Data presented as n (%), mean ± standard deviation, or median [interquartile range].

**Table 3:** CT and echocardiographic imaging follow-up by conduit type.

	Pre-Assembled AVC		Hand-Sewn AVC	
CT Aortic Diameters	First Post-Op CT (n = 100)	Latest Follow-Up CT (n = 42)	First Post-Op CT (n = 44)	Latest Follow-Up CT (n = 21)
Time post-op, days	203 [122–244]	576 [244–722]	65 [41–142]	519 [153–769]
**Annulus, coronal**	25.62 ± 2.87	26.07 ± 3.02	22.60 ± 3.19	22.06 ± 3.59
**SoV, coronal**	37.63 ± 3.27	37.97 ± 3.38	31.37 ± 6.89	31.11 ± 6.54
**SoV, axial**	38.80 ± 3.65	39.84 ± 4.20	32.08 ± 6.49	31.83 ± 6.87
**SoV, sagittal**	38.56 ± 3.59	38.98 ± 3.61	32.09 ± 6.47	31.74 ± 5.63
**DA, coronal**	28.21 ± 3.65	28.09 ± 3.46	25.97 ± 4.14	25.58 ± 3.57
**DA, axial**	29.44 ± 3.71	29.11 ± 3.35	27.03 ± 4.34	26.46 ± 3.31
**DA, sagittal**	29.28 ± 3.68	29.39 ± 2.95	27.30 ± 4.35	26.91 ± 4.49
**Echo Variables**	**First Post-Op echo** **(n = 117)**	**Latest Follow-Up Echo** **(n = 93)**	**First Post-Op echo** **(n = 42)**	**Latest Follow-Up Echo** **(n = 27)**
Time post-op, days	6 [5–24]	292 [195–626]	60 [6–273]	522 [326–770]
**Aortic valve flow**				
** Peak velocity, m/s**	1.85 ± 0.37	1.90 ± 0.33	2.38 ± 0.61	2.21 ± 0.50
** Peak gradient, mmHg**	14.13 ± 5.69	14.59 ± 5.33	23.94 ± 11.92	20.19 ± 9.19
** Mean gradient, mmHg**	7.66 ± 3.02	8.08 ± 2.87	13.46 ± 6.24	11.51 ± 5.16
**EOA, cm^2^**	2.44 ± 0.76	2.13 ± 0.65	1.78 ± 0.64	1.81 ± 0.33
**Indexed EOA, cm^2^/m^2^**	1.16 ± 0.34	1.05 ± 0.31	0.87 ± 0.33	0.83 ± 0.18
**LVEF, %**	57.02 ± 9.94	58.27 ± 7.46	58.63 ± 11.28	57.38 ± 14.25
**LVEDV, mL**	150.46 ± 49.90	136.66 ± 38.61	137.39 ± 52.87	118.57 ± 37.33
**LVESV, mL**	68.56 ± 39.27	57.03 ± 20.50	65.41 ± 53.84	50.90 ± 24.84

Data presented as mean ± standard deviation or median [interquartile range]. Diameter measurements are reported in mm unless otherwise stated. Abbreviations: AVC, aortic valved conduit; CT, computed tomography; SoV, sinus of Valsalva; DA, distal anastomosis; EOA, effective orifice area; LVEF, left ventricular ejection fraction; LVEDV, left ventricular end diastolic volume; LVESV, left ventricular end systolic volume.

**Table 4:** Linear mixed-effects model estimates for baseline, yearly change, and interaction effects between pre-assembled and hand-sewn aortic valved conduits.

Variable	Baseline Difference (Pre-Assembled vs. Hand-Sewn)		Yearly Change (Hand-Sewn)		Difference in Yearly Change (Pre-Assembled vs. Hand-Sewn)	
	Estimate	*p*-Value	Estimate	*p*-Value	Estimate	*p*-Value
**CT aortic diameters**						
**Annulus, coronal, mm**	3.11 (1.88 to 4.33)	<0.001	0.10 (−0.53 to 0.73)	0.76	−0.12 (−0.96 to 0.73)	0.78
**SoV, coronal, mm**	6.12 (4.34 to 7.91)	<0.001	0.02 (−0.75 to 0.79)	0.96	−0.25 (−1.28 to 0.78)	0.64
**SoV, axial, mm**	5.93 (4.06 to 7.79)	<0.001	0.18 (−0.61 to 0.98)	0.65	0.56 (−0.52 to 1.64)	0.31
**SoV, sagittal, mm**	6.74 (4.96 to 8.52)	<0.001	0.69 (−0.10 to 1.47)	0.092	−1.04 (−2.10 to 0.03)	0.059
**DA, coronal, mm**	2.34 (0.84 to 3.83)	0.003	−0.19 (−0.91 to 0.52)	0.59	−0.26 (−1.21 to 0.69)	0.59
**DA, axial, mm**	2.39 (0.90 to 3.88)	0.002	0.10 (−0.61 to 0.81)	0.78	−0.13 (−1.07 to 0.82)	0.79
**DA, sagittal, mm**	2.22 (0.68 to 3.76)	0.005	−0.06 (−0.88 to 0.75)	0.88	−0.24 (−1.30 to 0.81)	0.65
**Echo variables**						
**Peak velocity, m/s**	−0.53 (−0.70 to −0.36)	<0.001	−0.11 (−0.20 to −0.02)	0.015	0.14 (0.03 to 0.24)	0.012
**Peak gradient, mmHg**	−9.06 (−12.14 to −5.98)	<0.001	−1.61 (−3.22 to 0.00)	0.051	1.91 (−0.05 to 3.87)	0.057
**Mean gradient, mmHg**	−5.32 (−6.85 to −3.79)	<0.001	−0.73 (−1.49 to 0.02)	0.059	0.95 (0.03 to 1.86)	0.044
**EOA, cm^2^**	0.48 (0.05 to 0.92)	0.031	−0.03 (−0.31 to 0.24)	0.81	−0.15 (−0.46 to 0.15)	0.32
**Indexed EOA, cm^2^/m^2^**	0.27 (0.05 to 0.49)	0.018	−0.01 (−0.14 to 0.12)	0.90	−0.08 (−0.23 to 0.06)	0.26
**LVEF, %**	0.30 (−3.45 to 4.05)	0.88	1.09 (−0.72 to 2.91)	0.238	0.14 (−2.11 to 2.40)	0.901
**LVEDV, mL**	0.05 (−0.21 to 0.31)	0.72	−0.02 (−0.16 to 0.13)	0.84	0.02 (−0.16 to 0.21)	0.79
**LVESV, mL**	−0.09 (−0.36 to 0.18)	0.51	−0.13 (−0.27 to 0.01)	0.080	0.12 (−0.06 to 0.29)	0.19

*p*-values correspond to fixed-effect estimates from linear mixed-effects models. Data presented as estimates (95% confidence interval). Abbreviations: CT, computed tomography; SoV, sinus of Valsalva; DA, distal anastomosis; EOA, effective orifice area; LVEF, left ventricular ejection fraction; LVEDV, left ventricular end diastolic volume; LVESV, left ventricular end systolic volume.

## Data Availability

The data that support the findings of this study are available from the corresponding author upon reasonable request. The data are not publicly available due to patient privacy and institutional restrictions.

## Abbreviations

AVC	Aortic Valved Conduit
CABG	Coronary Artery Bypass Graft
CPB	Cardiopulmonary Bypass
CT	Computed Tomography
ECMO	Extracorporeal Membrane Oxygenation
EOA	Effective Orifice Area
TAVR	Transcatheter Aortic Valve Replacement
IQR	Interquartile Range
LVEF	Left Ventricular Ejection Fraction
